# Separately or Combined, LukG/LukH Is Functionally Unique Compared to Other Staphylococcal Bicomponent Leukotoxins

**DOI:** 10.1371/journal.pone.0089308

**Published:** 2014-02-20

**Authors:** Machi Yanai, Miguel A. Rocha, Anthony Z. Matolek, Archana Chintalacharuvu, Yasuhiko Taira, Koteswara Chintalacharuvu, David O. Beenhouwer

**Affiliations:** 1 Division of Infectious Diseases, Veterans Affairs Greater Los Angeles Healthcare System, Los Angeles, California, United States of America; 2 Emergency and Critical Care, St. Marianna University School of Medicine, Kawasaki, Kanagawa, Japan; 3 Department of Medicine, David Geffen School of Medicine, University of California, Los Angeles, California, United States of America; National Institutes of Health, United States of America

## Abstract

*Staphylococcus aureus* is a major human pathogen that elaborates several exotoxins. Among these are the bicomponent leukotoxins (BCLs), which include γ-hemolysin, Panton-Valentine leukocidin (PVL), and LukDE. The toxin components are classified as either F or S proteins, which are secreted individually and assemble on cell surfaces to form hetero-oligomeric pores resulting in lysis of PMNs and/or erythrocytes. F and S proteins of γ-hemolysin, PVL and LukDE have ∼70% sequence homology within the same class and several heterologous combinations of F and S members from these three bicomponent toxin groups are functional. Recently, an additional BCL pair, LukGH (also called LukAB) that has only 30% homology to γ-hemolysin, PVL and LukDE, has been characterized from *S. aureus*. Our results showed that LukGH was more cytotoxic to human PMNs than PVL. However, LukGH-induced calcium ion influx in PMNs was markedly attenuated and slower than that induced by PVL and other staphylococcal BCLs. In contrast to other heterologous BCL combinations, LukG in combination with heterologous S components, and LukH in combination with heterologous F components did not induce calcium ion entry or cell lysis in human PMNs or rabbit erythrocytes. Like PVL, LukGH induced IL-8 production by PMNs. While individual components LukG and LukH had no cytolytic or calcium influx activity, they each induced high levels of IL-8 transcription and secretion. IL-8 production induced by LukG or LukH was dependent on NF-κB. Therefore, our results indicate LukGH differs functionally from other staphylococcal BCLs.

## Introduction

*Staphylococcus aureus* secretes a large number of proteins and peptides, including the bicomponent leukotoxins (BCLs), that target the host cell membrane. These toxins consist of two separately secreted components, S and F, named for their speed of elution by cationic exchange (slow versus fast), which do not have significant cytolytic activity individually. The ∼32 kDa S component binds target cell membranes and then assembles with the ∼38 kDa F component, leading to formation of pores on the cell membrane [1], [2]. Five BCL operons plus several variants have been identified in *S. aureus*; four are found in strains that cause human disease: γ-hemolysin, Panton-Valentine leukocidin (PVL), LukDE and the recently described LukGH (also termed LukAB) [3]. Prior studies have shown that S components of γ-hemolysin, PVL, LukDE (HlgA, HlgC, LukS-PV or LukE) determine cell specificity and can assemble with other F components (HlgB, LukF-PV or LukD) leading to toxin pairs with various properties [2]. The activity of LukG in combination with heterologous F components or LukH in combination with heterologous S components has not been reported.

Neutrophils (polymorphonuclear cells, PMNs), which are the first line of defense against *S. aureus* infections, are a primary target of BCLs. Binding of a class S component to the cell membrane is a prerequisite for secondary binding of a class F component [4], which in combination induce rapid calcium channel-mediated influx of calcium ions into PMNs [5]. The S and F proteins form a heterologous octameric pore in the membrane of target cells [6], [7]. Furthermore, inflammatory cytokines, such as interleukin-8 (IL-8) and IL-1β, are expressed by PMNs after leukotoxin exposure [8], [9]. γ-hemolysin and LukDE also have activity against red blood cells (RBCs) [10].

Recently, an additional BCL pair, LukG/LukH (also called LukB/LukA) was identified from both methicillin-sensitive and methicillin-resistant *S. aureus* (MSSA and MRSA, respectively) strains [11], [12]. While related to other BCLs, LukG and LukH show distinctly less homology to other members of their respective classes. The amino acid sequence of LukG is 36–37% identical to other F components (HlgB, LukF-PV) and that of LukH is 26–28% identical to other S components (HlgA, HlgC, LukS-PV) [11], [12].

In this report, we used individual recombinant proteins to compare the characteristics of LukG and LukH to other staphylococcal BCLs. We identified differences with respect to calcium ion influx into PMNs, PMN lysis, hemolysis and the ability to form active toxin pairs with heterologous components. In addition, we found that the single components LukG and LukH induced IL-8 production in a NF-κB dependent manner. Thus, these proteins join LukS and HlgB as members of the staphylococcal BCL family described to have individual activity [13], [14]. Our understanding of the role these toxins in staphylococcal infection continues to evolve. This study adds to our knowledge of the complex interactions and effects of this diverse family of exotoxins.

## Methods

### Recombinant Protein Production and Purification

LukF-PV, LukS-PV, HlgA, HlgB, HlgC, LukG and LukH were amplified from *S. aureus* V8 (ATCC 49775) and LukD and LukE were amplified from strain Newman using the primers listed in Table 1. The primers for LukG and LukH were designed based on published sequences [11]. Amplified PCR products were ligated into pET151D-TOPO directional prokaryotic expression vector (Invitrogen) and DNA sequences were confirmed. Plasmids were transformed into BL21 star (DE3) *Escherichia coli* (Invitrogen) and grown in LB media with carbenicillin (50 µg/mL) at 37°C in the presence of 0.5 mM isopropyl β-D-1-thiogalactopyranoside (IPTG). Bacteria were collected by microcentrifugation at 10,000 rpm for 10 min.

**Table 1:** Primers used for plasmid construction and reverse-transcription PCR.

	Forward primer (5′-3′)	Reverse primer (5′-3′)
**LukF-PV**	CACCGCTCAACATATCACACCTGT	TTAGCTCATAGGATTTTTTTCCTTAG
**LukS-PV**	CACCGAATCTAAAGCTGATAACAAT	TCAATTATGTCCTTTCACTTTAATTTC
**HlgA**	CACCATGGAAAATAAGATAGAAGATA	CTTAGGTGTGATGCTTTTAATTTTTAC
**HlgB**	CACCATGGCTGAAGGCAAAATCACACC	TTTATTGTTTTCAGTTTCTTTTGTATC
**HlgC**	CACCATGAAAGCTGCCAATGATACTGAAG	ATTCTGTCCTTTCACTTTGATTTC
**LukD**	CATGTAGGATCCTTAATATCACACCTAAAAGAGAGAAAAAAGTAG	CATGTAGAGCTCTTATACTCCAGGATTAGTTTCTTTAGAATCC
**LukE**	CATGTAGAATTCTTAATATTGAAAATATTGGTGATGGTGCTG	CATGTAGAGCTCTTAATTATGTCCTTTCACTTTAATTTCGTGT
**LukG**	CACCGCTACTTCATTTGCAAAG	TTATTTCTTTTCATTATCATTAAGTACTTTTAC
**LukH**	CACCACTCAAGCAAATTCAGCT	TTATCCTTCTTTATAAGGTTTATTGTCATC
**IL-8**	CTCTTGGCAGCCTTCCTGATTTCT	ACCCTCTGCACCCAGTTTTCCTTG
**β-actin**	ACCCACACTGTGCCCATCTA	CGGAACCGCTCATTGCC

To purify LukF-PV and LukS-PV, pelleted bacteria were lysed in a French pressure cell (Thermo Scientific) at 5000 pounds per square inch. The resultant lysate was mixed with nickel resin beads (Qiagen) for 12 h at 4°C. After washing the beads first with 15 mM and then with 40 mM imidazole buffers, samples were eluted with 250 mM imidazole buffer. The eluate was dialyzed against phosphate buffered saline (PBS). To purify HlgA, HlgB, HlgC, LukD, LukE, LukG and LukH, bacterial pellets were resuspended in bacterial protein extraction reagent (B-PER, Thermo Scientific) following the manufacturer’s instructions. After centrifugation, pellets were dissolved in 8 M urea buffer containing 10 mM tris, 50 mM sodium phosphate (pH 8.0) and then applied to nickel resin beads (Qiagen), mixed for 20 min at room temperature. After washing with pH 6.3 and pH 5.9 urea buffers, proteins were eluted using pH 4.5 urea buffer. The eluate was dialyzed against 500 mM L-arginine buffer containing 200 mM sodium chloride (NaCl), 50 mM tris, 1 mM ethylenediamine tetraacetic acid (EDTA) buffer (pH 8.0). The polyhistidine tag was removed from the recombinant proteins using AcTEV protease (Invitrogen). Finally, all recombinant proteins were subjected to LPS removal using DetoxiGel columns (Thermo Scientific). Proteins were analyzed for purity and proper size by Coomassie-stained SDS-PAGE and quantitated by bicinchoninic acid (BCA) assay (Pierce). To demonstrate proteins and not LPS or some other contaminant were responsible for IL-8 release by PMNs, recombinant LukG and LukH (500 µg) were individually treated with chymotrypsin 20 µg in 1 mL HBSS for 24 hr at 37°C. Complete digestion was verified by SDS-PAGE. A small quantity of protease for an extended incubation period was used to minimize potential carryover to experimental samples. Protease inactivity in the digested samples was confirmed by exposing 5 µg bovine serum albumin (BSA) to the digest mixtures for 24 hr at 37°C followed by visualization of intact BSA on SDS-PAGE.

### Preparation of Human Neutrophils

Approval for human studies was obtained from the VA Greater Los Angeles Institutional Review Board (IRB). All acquisition and analysis of human samples was performed at VA Greater Los Angeles and not at any other institution. Participants provided written consent to participate in the study. The consent process and form was reviewed and approved by the IRB. After informed consent, peripheral blood obtained from healthy donors was mixed with an equal volume of 3% dextran/0.9% NaCl solution. The leukocyte-rich plasma was separated after 20 min incubation at room temperature and cells were pelleted by centrifugation at 1000 rpm for 10 min at 5°C. Resuspended cells in 0.9% NaCl were layered over 10 mL Ficoll-Paque (GE Healthcare) and then spun at 1400 rpm at 20°C for 40 min. PMNs were isolated by centrifugation at 1000 rpm at 5°C for 6 min after lysing RBCs by 0.2% NaCl and 1.6% NaCl. Purified PMNs were suspended in Hank’s balanced salt solution (HBSS). PMN purity (>95%) was confirmed with Diff Quick (Thermo Scientific) staining. To examine the role of calcium channels in calcium ion entry, in a set of experiments, methoxyverapamil (Sigma) was added at 100 µM or 400 µM 60 min prior to addition of BCL components.

### Calcium Assay

PMNs were resuspended in HBSS without calcium and magnesium at 5×10^6^/mL. PMNs were incubated with 4 µM Fluo-4 AM (Invitrogen) and 0.1% Pluronic F-127 (Invitrogen) for 30 min at room temperature in the dark. The cells were washed once with HBSS and collected by centrifugation at 1500 rpm for 5 min. PMNs were then incubated in HBSS with 250 mM probenecid (Invitrogen) for another 30 min at 37°C. After the incubation period, 2×10^6^ cells/mL were mixed with BCL components in a 96-well plate in the presence of 1.1 mM CaCl_2_. Fluorescence was measured every minute at an emission wavelength of 520 nm and excitation wavelength at 485 nm using a Synergy 2 plate reader (BioTec). Plates were kept at 37°C and shaken every 30 sec. The maximal and minimal fluorescence were determined by incubation with 1% Triton X-100 and 1 mM ethylene glycol tetraacetic acid (EGTA), respectively. Percent Fluo-4 fluorescence intensity was calculated according to the following formula: 100× (F_observed_−F_minimum_)/(F_maximum_−F_minimum_). All samples were assayed in triplicate.

### Cytotoxicity Assay

PMN (1×10^6^ cells) lysis was determined by measuring lactate dehydrogenase (LDH) using the Cytotoxicity Detection Kit (Roche Applied Sciences) as described previously [15]. The maximal LDH release was determined by incubation with 1% Triton X-100. All samples were assayed in duplicate. Based on measured absorbance, percent LDH release was calculated according to the following formula: 100× (A_sample_−A_background_)/(A_1% triton_−A_background_).

### Determination of Hemolytic Activity

Fifty µL of 5% rabbit erythrocytes were incubated with recombinant BCLs or α-toxin (Sigma) for 1 hour at 37°C in a 96-well round-bottom plate in a final volume of 100 µL. Intact cells were pelleted by centrifugation, 50 µL of supernatants were transferred to a new plate and absorbance at 450 was measured. Complete lysis of erythrocytes was induced by 20 µg/mL α-toxin. Hemolysis was calculated using the following formula: percent hemolysis = 100× (A_sample_−A_control_)/(A_α-toxin_−A_control_). A_sample_ and A_control_ are the absorbance by the sample with and without leukotoxin, respectively. A_α-toxin_ is the absorbance observed with 20 µg/mL α-toxin. All samples were assayed in duplicate.

### IL-8, TNF-α Assays

PMNs were resuspended in HBSS containing 1.3 mM CaCl_2_ and 0.5 mM MgCl_2_ at a final concentration of 2×10^6^/mL. Cells were incubated with BCL components at 37°C with 5% CO_2_ for 2–8 hours in 100 µL in 96-well cell culture plates. In the NF-κB inhibition assay, some PMNs were treated with 50 µM of (E)-3-(4-Methylphenylsulfonyl)-2-propenetrile (Bay11-7082, an inhibitor of IκBα phosphorylation, Sigma) for 1 h before addition of BCLs. IL-8 and TNF-α in cell supernatants were measured by ELISA using BD OptEIA human IL-8 ELISA set and human TNF-α DuoSet (R&D Systems) following the manufacturer’s instructions. All samples were assayed in duplicate.

### Reverse Transcription-PCR (RT-PCR)

mRNA was isolated from 8×10^4^ PMNs using mRNA Catcher PLUS (Invitrogen) and cDNA was synthesized using oligo-dT primer according to the protocol provided by the manufacturer. Two µL of cDNA was amplified with relevant primer combinations (Table 1) via PCR with 1 min denaturation at 95°C, 1 min annealing at 57°C (for β-actin) or 62°C (for IL-8), and 1 min elongation at 72°C. Amplification was continued for 40 cycles. PCR products were visualized by electrophoresis on 1.5% agarose gels containing ethidium bromide.

### Statistical Analysis

Data were analyzed using Prism 5.0 (GraphPad, La Jolla, CA, USA). Statistically significant differences were evaluated by unpaired *t*-test or one-way ANOVA followed by Tukey’s post-test. *P* value <0.05 was considered significant.

## Results

### LukGH Induces Calcium Ion Entry into PMNs with Attenuated Kinetics

**Figure 1:**
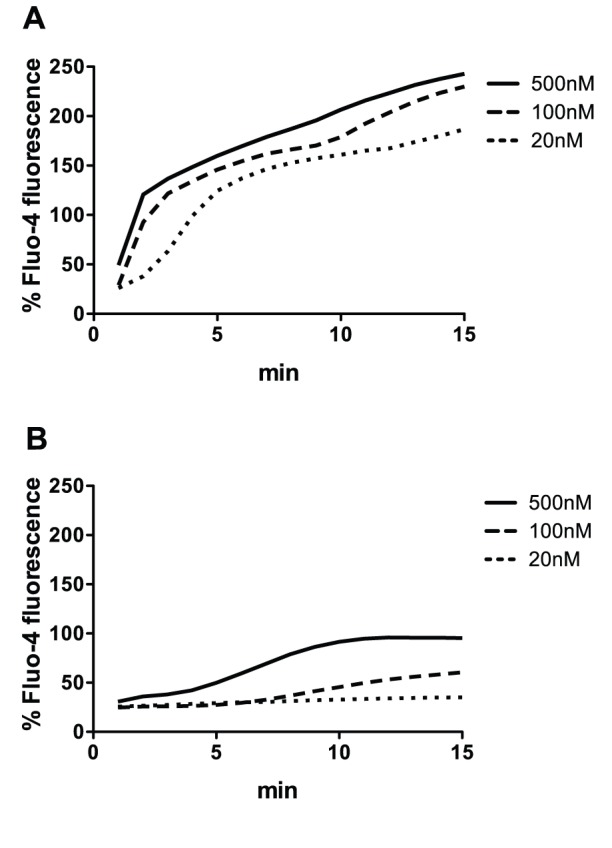
Calcium ion entry into human PMNs in presence of PVL (A) and LukGH (B) at the indicated concentrations. Leukotoxins were mixed with 2×10^6^/mL PMNs loaded with 4 µM Fluo-4 in presence of 1.1 mM CaCl_2_. Fluorescence intensity was recorded immediately and then every minute. Percent Fluo-4 fluorescence was calculated according to the formula described in the methods section by using 1% Triton X-100 to estimate maximal fluorescence and 1 mM EGTA to determine minimal fluorescence. The results represent the mean of four independent experiments.

**Figure 2:**
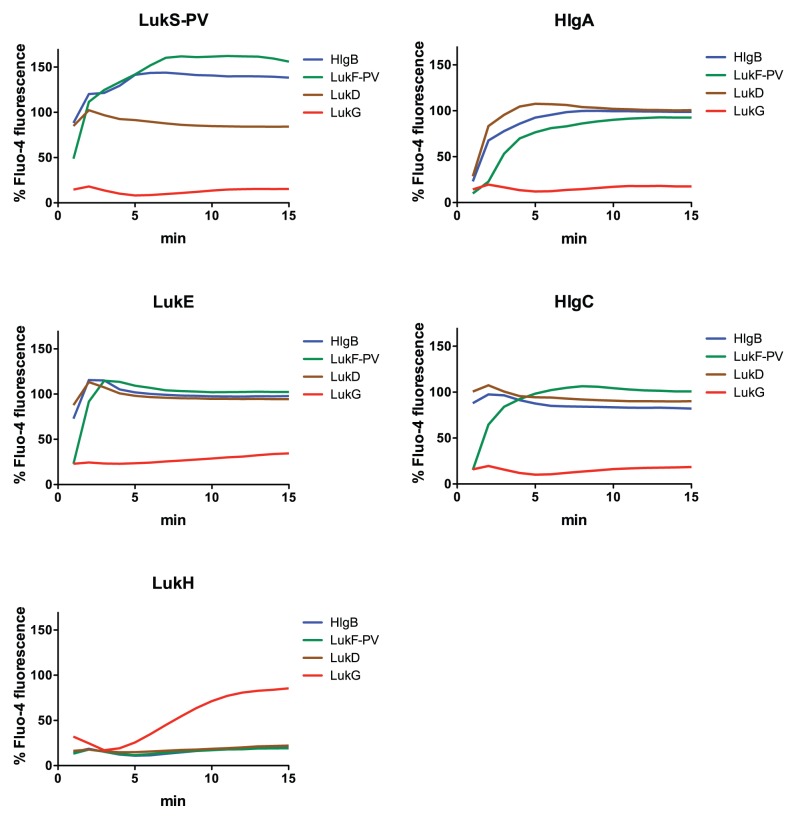
Calcium ion entry into human PMNs by combinations of S and F components from LukGH, PVL, LukDE and γ-hemolysin. Calcium flux was determined as described in Figure 1. Each panel shows a particular S component (header) as mixed with various F components (LukG: red, LukF-PV: green, LukD: brown, HlgB: blue). The concentration of all proteins was 500 nM. The results shown are the mean of three independent experiments.

After the binding of staphylococcal BCLs to human PMNs, there is a rapid increase of intracellular free calcium ion concentration, which is followed by pore formation and cell lysis [5], [16]. To investigate calcium ion entry by LukGH, we mixed recombinant BCLs with Fluo-4 AM-loaded human PMNs and measured fluorescence intensities. While no calcium entry was observed when cells were incubated with either LukG or LukH alone (data not shown), LukG+LukH induced calcium ion entry in a dose dependent manner with 50% maximal attained in 7.5±0.6 minutes in the presence of 500 nM LukGH (Figure 1B). In contrast, PVL induced calcium ion entry much more rapidly with 50% maximal attained in 2.3±0.2 minutes in the presence of 500 nM PVL (Figure 1A). PVL was a much more potent inducer of calcium ion entry, with 20 µM showing significantly greater activity than 500 µM LukGH. At 20 µM, LukGH did not induce calcium ion entry into PMNs during the 15 min observation period. Other BCL pairs, including HlgBA, HlgBC and LukDE induced rapid calcium entry similar to PVL (Figure 2). Thus, LukGH induced calcium influx but with attenuated kinetics and potency in human PMNs compared to other staphylococcal BCLs.

**Figure 3:**
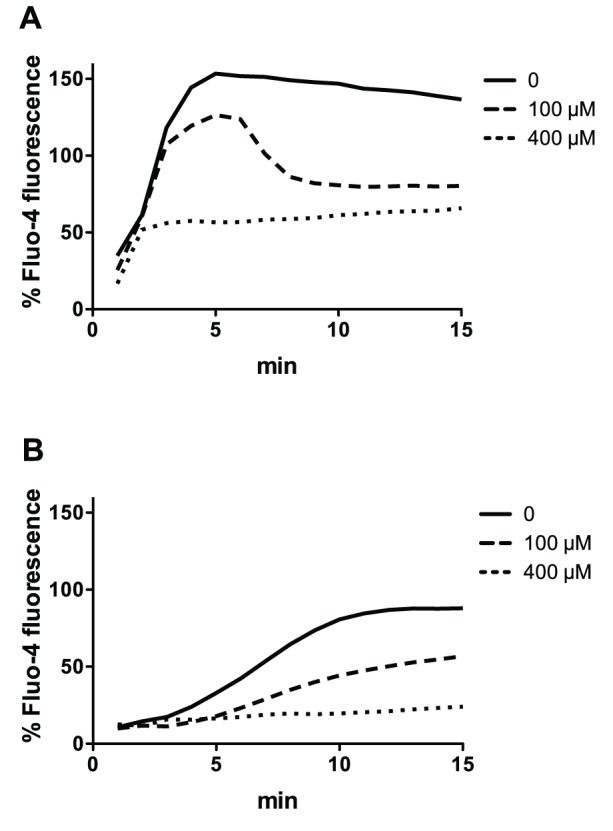
The effect of calcium channel blockade on calcium ion entry into human PMNs in presence of 20(A) or 500 nM LukGH (B). PMNs (2×10^6^/mL) were loaded with 4 µM Fluo-4 in presence of 1.1 mM CaCl_2_ and then exposed to the indicated concentration of methoxyverapamil for 60 min prior to adding PVL or LukGH. Calcium flux was then determined as described in Figure 1. The results represent the mean of three independent experiments.

Calcium channel blockers, such as methoxyverapamil, can prevent calcium ion influx in PMNs induced by PVL or HlgA/HlgB without inhibiting pore formation [5]. To determine whether the calcium entry mediated by LukGH similarly occurred via calcium channels, PMNs were incubated with methoxyverapamil for one hour prior to the addition of LukGH (Figure 3B) or PVL as control (Figure 3A). Calcium ion entry was inhibited in a dose-dependent fashion by methoxyverapamil suggesting that for both toxin pairs this ion flux was mediated by calcium channels.

### LukGH is a Potent Cytolytic Agent for Human PMNs, but Lacks Hemolytic Activity

**Figure 4:**
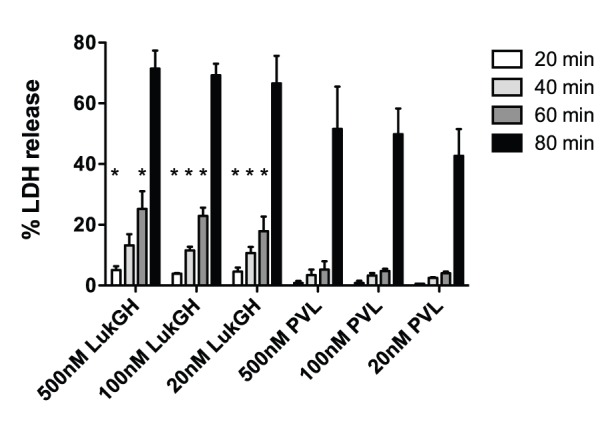
Cytolytic activities of LukGH and PVL in human PMNs. PMNs (1×10^6^/mL) were incubated with indicated concentrations of LukGH or PVL for 20–80 min and LDH concentration in the supernatant was measured using the Roche Cytotoxicity Detection Kit. Percent LDH release was calculated according to the formula described in the methods section by using 1% Triton X-100 to estimate maximal LDH release. The results represent the mean of three independent experiments ± SEM. * indicates statistical significance from PVL at the same concentration and time.

**Figure 5:**
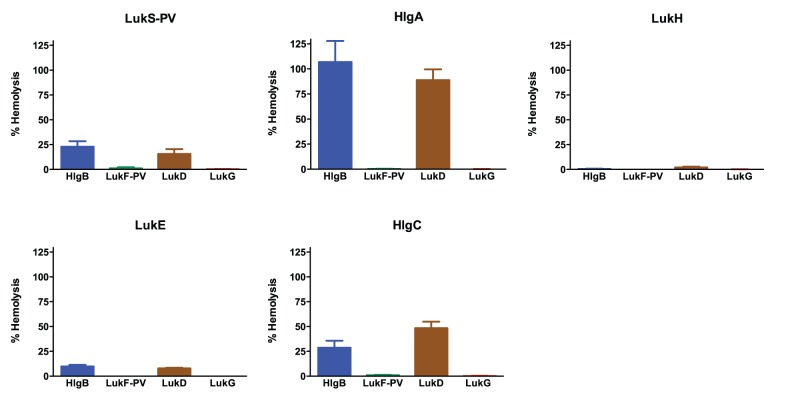
Hemolytic activity by combinations of S and F components from LukGH, PVL, LukDE, and γ-hemolysin. Five percent rabbit RBCs were incubated with BCL pairs at 500°C in a 96-well round-bottom plate. After pelleting intact cells, supernatants were transferred to a new plate and the absorbance at 450 nm was measured. Hemolysis was calculated according to the formula described in the methods section by using 20 µg/mL *S. aureus* α-toxin to determine complete RBC lysis. The results shown are the mean of three independent experiments ± SEM. Each panel shows a particular S component (header) as mixed with various F components (LukG: red, LukF-PV: green, LukD: brown, HlgB: blue).

To determine cytolytic activity, human PMNs were incubated with 20, 100 and 500 nM LukGH or PVL and the LDH released by lysed cells was measured (Figure 4). Prior studies examining the activity of γ-hemolysin and PVL at concentrations ranging from 2–200 nM have shown that native leukotoxins are highly cytolytic for PMNs at 2–20 nM [4], [5], [17]. Recombinant BCLs may have less activity than their native counterparts [18]. Studies showing activity of individual BCL components used concentrations in the 300 nM range [13], [14]. Thus we examined concentrations in the range of 20–500 nM. By 80 min, both LukGH and PVL induced similar amounts of LDH release (Figure 4). However, in the first 60 min, LukGH was significantly more toxic to PMNs than PVL. These results suggest that while both LukGH and PVL are cytolytic to human PMNs, the cytolytic activity of LukGH is more potent than PVL in the first 60 min. As expected, single components (LukF-PV, LukS-PV, LukG or LukH) did not cause LDH release (data not shown). Leukotoxins other than PVL demonstrate hemolytic activity with HlgBA being the most potent toxin pair in this regard. LukGH did not show hemolytic activity (Figure 5).

### LukG and LukH do not Form Active Toxin Pairs with Heterologous Leukotoxin Components

**Figure 6:**
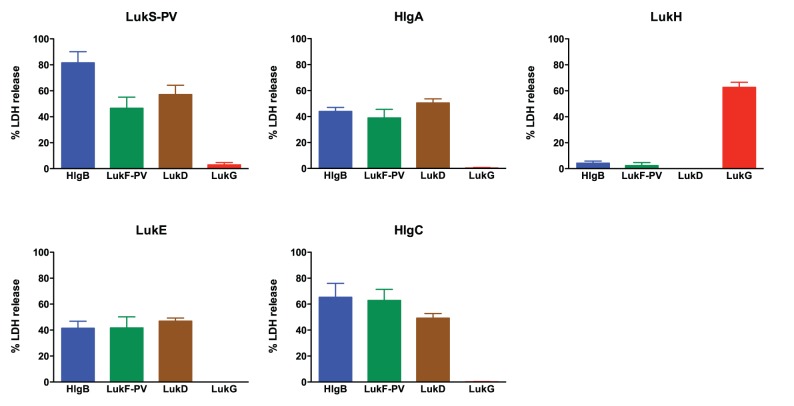
Cytolytic activity of combinations of S and F components from LukGH, PVL, LukDE, and γ-hemolysin. LDH release was determined as described in Figure 4. Each panel shows a particular S component (header) as mixed with various F components (LukG: red, LukF-PV: green, LukD: brown, HlgB: blue). The concentration of all proteins was 500 nM. The results shown are the mean of three independent experiments ± SEM.

Prior studies have shown that the combination of certain heterologous F and S components have cytolytic activity and can induce calcium influx [10], [19]–[21]. To determine if LukG and LukH can combine with other leukotoxins to form active toxin pairs, we determined the activity of 1) LukG when combined with heterologous S components (LukS-PV, HlgA, HlgC and LukE), and 2) LukH combined with heterologous F components (LukF-PV, HlgB and LukD). All combinations of F components and S components of PVL, γ-hemolysin and LukDE induced calcium ion entry and cell lysis (Figures 2 and 6). On the contrary, combinations of LukG with heterologous S components did not lead to calcium ion entry or cytotoxicity (Figures 2 and 6). The same was true of LukH when combined with heterologous F components. Individual toxin components did not show activity (data not shown).

Heterologous leukotoxin pairs have previously been shown to be hemolytic for rabbit RBCs (LukD+HlgA>HlgB+LukS-PV>LukF-PV+HlgA) [10], [19]. Consistent with these reports, we found that LukD+HlgA demonstrated the most potent hemolytic activity and HlgB+LukS-PV showed moderate activity for rabbit RBCs (Figure 5). In contrast to prior findings, we also found HlgC+LukD to have significant activity and HlgB+LukE and LukD+LukS-PV showed mild-moderate hemolytic activity (Figure 5). Furthermore, LukF-PV+HlgA did not induce hemolysis. All combinations of LukG with heterologous S components or LukH with heterologous F components did not induce detectable hemolysis. Individual components did not show hemolytic activity (data not shown). Table 2 summarizes our findings concerning heterologous leukotoxin activity in PMN cytotoxicity and calcium flux and hemolysis.

**Table 2:** Characteristics of heterologous toxin combinations.

S component	F component	PMN lysis	PMN Ca^2+^ influx	Hemolysis
**HlgA**	HlgB	++	+++	+++
	LukF-PV	++	++	−
	LukD	++	+++	+++
	LukG	−	−	−
**HlgC**	HlgB	+++	+++	+
	LukF-PV	+++	++	−
	LukD	++	+++	++
	LukG	−	−	−
**LukS-PV**	HlgB	++++	+++	+
	LukF-PV	++	+++	−
	LukD	++	+++	+
	LukG	−	−	−
**LukE**	HlgB	++	+++	+
	LukF-PV	++	+++	−
	LukD	++	+++	+
	LukG	−	−	−
**LukH**	HlgB	−	−	−
	LukF-PV	−	−	−
	LukD	−	−	−
	LukG	+++	+	−

### LukG and LukH Individually are Potent Inducers of IL-8 Expression by Human PMNs

**Figure 7:**
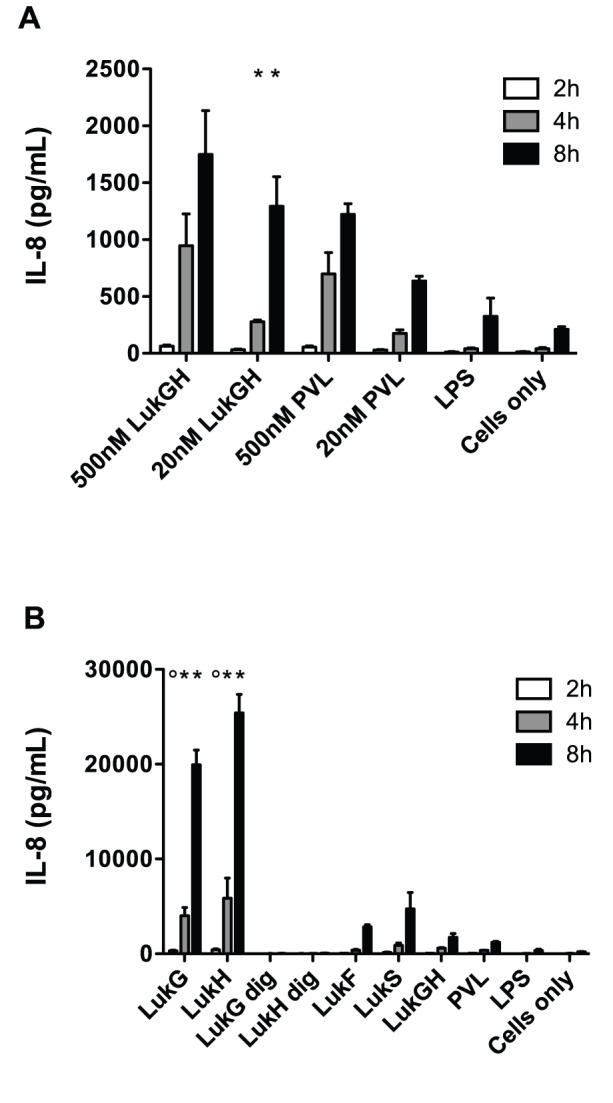
IL-8 production by PMNs treated with LukGH or PVL. PMNs (2×10^6^/mL) were incubated in the presence or absence of (A) indicated concentrations of LukGH, PVL, LPS (50 µg/mL) and (B) single leukotoxin components, protease-digested single components (LukG dig and LukH dig), LukGH, PVL (all 500 nM) or LPS (50 µg/mL) for 2–8 h. IL-8 levels in the supernatants were measured by ELISA using recombinant IL-8 as a standard. The results represent the mean of three independent experiments ± SEM. In (A), * indicates statistical significance compared to PVL. In (B), * indicates significance compared to both LukF-PV and LukS-PV and **°** indicates significance to LukF-PV only.

PVL has been reported to induce IL-8 release by PMNs and monocytes [8], [22], [23]. To determine if LukGH similarly induces IL-8, we incubated human PMNs with leukotoxins for 2–8 hours and measured IL-8 in the supernatants by ELISA. While, both PVL and LukGH induced IL-8 from PMNs in a time and dose dependent manner, LukGH induced 2–3 fold more IL-8 production than PVL (Figure 7A). However, cells incubated with toxin pairs at 500 nM were observed by microscopy to be fully lysed by 4 hrs, and most cells appear to be lysed by 30–45 min (data not shown). When cells were incubated with LukG or LukH individually, they produced 10-fold or greater IL-8 than when both LukG and LukH were present (Figure 7B). In addition, the individual components LukF-PV and LukS-PV also induced 2–4 fold more IL-8 than when both LukF-PV and LukS-PV were present, but over 6-fold less than when the individual components LukG and LukH were present. Other individual components, including LukD, LukE, HlgA, HlgB and HlgC did not induce significant IL-8 (data not shown). We confirmed that none of the single components caused cell lysis after 8 hours by both LDH release assay and light microscopy.

Previous reports indicated that the presence of lipopolysaccharide (LPS) could induce IL-8 by human PMNs [24], [25]. To determine if the presence of small amounts of contaminating LPS in our recombinant proteins could be responsible for the high levels of IL-8 observed in culture supernatants, we incubated human PMNs with LPS from multiple sources. The levels of IL-8 produced in presence of 50 µg/mL LPS was marginally more than media alone and insignificant compared to when PVL was present (Figure 7B), indicating that LPS, if present in any significant amounts in our recombinant proteins, was not responsible for the IL-8 production by human PMNs. In addition, LukG and LukH were treated with chymotrypsin and complete digestion was confirmed by SDS-PAGE. The digested components did not induce IL-8 (Figure 7B). These results demonstrated that IL-8 induction was due to the toxin component and not to LPS and/or another contaminant. Thus, our results suggest that individually, LukG and LukH are potent inducers of the proinflammatory cytokine IL-8 by human PMNs.

### LukG and LukH Increase Production of IL-8 mRNA

**Figure 8:**
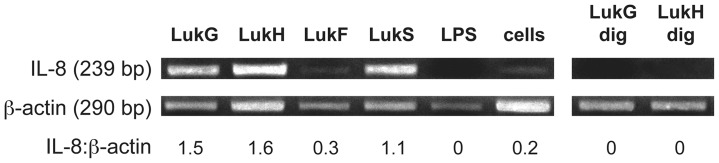
Induction of IL-8 mRNA by individual components of LukGH or PVL. PMNs were incubated for 1(cells) or with 500 nM LukG, LukH, LukF-PV, LukS-PV, protease-digested LukG (LukG dig) or LukH (LukH dig), or 50 µg/mL LPS, and total mRNA was isolated from cell lysate. cDNA was synthesized and the DNA was amplified using IL-8 and β-actin specific primers and PCR. The ratio of IL-8 transcripts to β-actin transcripts is shown below the gel image.

LukG and LukH could increase the amount of IL-8 detected in the supernatant of exposed PMNs by increasing: 1) IL-8 secretion, 2) translation from existing IL-8 mRNA transcripts or 3) the amount of IL-8 mRNA. To establish whether IL-8 mRNA transcripts were increased, we determined relative amounts of IL-8 mRNA in human neutrophils exposed to individual leukotoxin components using RT-PCR (Figure 8). LukG and LukH increased the production of IL-8 specific mRNA by 8-fold over untreated cells in 1 hour. In addition, LukS-PV induced IL-8 production by 6-fold. LPS (50 µg/mL) treatment had no effect on IL-8 production by human PMNs in this time period. LukG and LukH treated with proteases did not induce IL-8 mRNA. These results suggest that LukG, LukH and LukS-PV induce IL-8 in human PMNs by rapidly increasing the production and/or increasing the stability of IL-8 mRNA.

### LukG and LukH Induce IL-8 Production by Activating NF-κB

**Figure 9:**
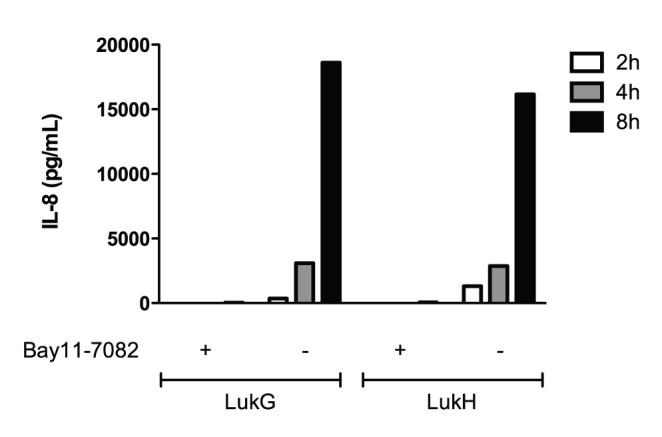
LukG and LukH induction of IL-8 production by PMNs pretreated with an inhibitor of NFκB. PMNs (2×10^6^/mL) were pretreated with Bay11-7082 (50 µM) for 1 h and then mixed with 500 nM LukG or LukH for 2–8 h. IL-8 levels in the supernatants were measured by ELISA.

NF-κB is a key transcriptional activator of IL-8 expression in human PMNs [26]. To determine if NF-κB is involved in the induction of IL-8 by LukG and LukH, human PMNs were pretreated with 50 µM of Bay11-7032, an inhibitor of IκBα phosphorylation that is nontoxic to PMNs [27], followed by incubation with either LukG or LukH for 2–8 h. IL-8 production was completely inhibited by Bay11-7082 (Figure 9). These results suggest that LukG and LukH induce the secretion of IL-8 by increasing the production of IL-8 mRNA in a NF-κB dependent manner.

In the presence of TNF-α, which is secreted by activated PMNs [28], the secretion of IL-8 is amplified and sustained [29]. However, TNF-α was not detected at significant levels in the cell supernatant from PMNs exposed to PVL, LukGH, or the individual toxin components LukG and LukH (data not shown).

## Discussion

*S. aureus* is a versatile pathogen that elaborates a diverse arsenal of virulence factors. Amongst these are the bicomponent leukotoxins (BCLs): γ-hemolysin, PVL, and LukDE. Several earlier studies have looked at the biochemistry and activity of these toxins (reviewed in [2]) defining their: 1) cytotoxic potential, 2) ability to induce calcium entry and release of inflammatory molecules in PMNs, and 3) ability to form active heterologous toxin pairs. Over the past few years, additional staphylococcal leukotoxins have been identified, including the phenol soluble modulins and LukGH. Initial studies of LukGH defined it as a toxin pair encoded in the staphylococcal genome, expressed by most staphylococcal strains and having cytotoxic activity against PMNs, macrophages and dendritic cells [11], [12]. In this study, we have further examined the effects of LukGH compared to other staphylococcal BCLs and determined that it has significantly different activity with respect to calcium ion entry and IL-8 production in human PMNs, as well as the lack of ability to form active heterologous toxin pairs (Table 3).

**Table 3:** Effects of the staphylococcal bicomponent leukotoxins [10], [30], [42], [43].

	PMNs	Monocytes	Lymphocytes	RBCs	IL-8	Ca^2+^ influx	Mouse	Rabbit	Macaque	Human
**LukGH**	++	+	−	−	+	Slow	−	+	++	++
**PVL**	++	++	−	−	+	Fast	−	++	−	++
**HlgBA** 1	++	+	+	++	+	Fast	ND^2^	ND	ND	++
**HlgBC** 1	++	+	−	+	+	Fast	ND	ND	ND	++
**LukDE**	+	ND	ND	+	?	Fast	ND	ND	ND	+

1 HlgA has also been called Hlg2, HlgB has been variously called Hlg1 or LukF and HlgC is also known as LukS.

2 ND no data available.

In general, S proteins bind to the PMN membrane followed by oligomerization with F protein molecules, after which there is rapid entry of calcium ions [16]. S and F proteins also form oligomeric pores on the cell membrane leading to cell lysis [16]. Compared to calcium ion influx, the process of functional pore formation is relatively slow, and there is a significant lag time between rapid calcium ion entry and pore formation as detected by ethidium ion entry [5], [17]. It has been established that the initial rapid calcium ion entry is independent of pore formation, occurs through activated calcium ion channels and is attenuated by calcium channel blockade [5], [21], [22]. Our results, indicating a rapid and high level of calcium ion influx immediately after the addition of γ-hemolysin, PVL or LukDE to PMNs, are consistent with published reports supporting a role for activated calcium ion channels in this process. In comparison, LukGH provoked a delayed and markedly reduced calcium ion influx. However, calcium channel blockade prevented calcium influx by LukGH, suggesting that LukGH operates via calcium channels, albeit much less effectively, like the other staphylococcal BCLs.

We chose to use recombinant proteins so that we could look at the effects of individual molecules without the concern that there might be contamination with other BCLs, as might occur if we used native proteins purified from *S. aureus*. However, a problem with using recombinant proteins produced in other bacteria is that secondary and tertiary structure may be different, leading to altered functional capabilities. Malachowa found that after 60 min there was 100% LDH release by 1000 nM native LukGH and ∼45% LDH release at 50 nM [30]. At 180 min there was 75% LDH release with 50 nM. We found that after 60 min, there was 25% and 20% LDH release at 500 nM and 20 nM recombinant LukGH, respectively. At 80 min, this increased to ∼70%. Thus, the native toxin components appear to be more potent, at least initially, compared to our recombinant LukGH. The recombinant proteins may also not interact as effectively in a heterologous manner. Thus, some caution should be applied when interpreting these results.

Cell specificity has been demonstrated for PVL, which targets PMNs and monocytes but not lymphocytes or RBCs [1], [2]. On the other hand, HlgBA demonstrates broad activity against multiple cell types, including RBCs [1], [2]. Studies of LukGH cell specificity suggest activity against PMNs, monocytes and dendritic cells [11], [12]. We have ascertained that LukGH does not have activity against RBCs, nor did LukG or LukH show hemolytic activity even when combined with heterologous components of γ-hemolysin. This suggests that LukGH binds to a specific cellular target, similar to PVL, rather than interacting directly with the membrane as does HlgBA [1], [2]. Consistent with these findings, CD11b has recently been identified as a cellular target of LukGH [31].

PVL-activated human PMNs, monocytes and macrophages release proinflammatory mediators, including IL-8 [8], [22], [23], [32]. Stimulated PMNs secrete IL-8 and at the same time they generate IL-8 as a cell-associated form that can be released after cell lysis [33], [34]. In our experiments, both PVL and LukGH induced IL-8 release by PMNs. Upon exposure to lytic concentrations of LukGH or PVL, PMNs are rapidly lysed and do not have time to elaborate significant IL-8. Thus, the majority of IL-8 detected by ELISA several hours following exposure to lytic concentrations of LukGH or PVL is likely to be preformed. After 8 hour exposure to single components LukG or LukH, PMNs produced several-fold higher levels of IL-8 than PVL, LukGH combined, or single components LukF-PV and LukS-PV. Our results indicate that LukG and LukH rapidly induce IL-8 production by increasing the level of IL-8 mRNA transcripts. In an autocrine manner, PMNs are also stimulated to produce IL-8 by other pro-inflammatory cytokines, such as TNF-α [29], [35]. However, TNF-α was not detected in the same culture supernatants incubated with LukG or LukH, indicating that IL-8 induction by LukG or LukH is not mediated by TNF-α. IL-8 is a potent chemoattractant for neutrophils and also has a wide range of other proinflammatory effects including degranulation from neutrophils, inducing expression of cell adhesion molecules and enhancing the adherence of neutrophils to endothelial cells [36]. Our studies suggest that LukG and LukH single components can activate PMNs to release IL-8, which could result in accumulation of PMNs at sites of inflammation. This may be advantageous to the host, since PMNs are the primary defense against *S. aureus* infections. However, this could also lead to increased killing of PMNs by leukotoxins secreted by *S. aureus*. Thus, in a paradoxical fashion, induction of IL-8 may be advantageous to the pathogen.

IL-8 induction by LukG or LukH was NF-κB dependent. It has been demonstrated that NF-κB is essential for IL-8 gene expression in a number of cells including PMNs [37]–[39]. Ma et al showed that PVL-induced inflammatory cytokine release is associated with the activation of NF-κB [23]. Transient increase of intracellular free calcium ion plays an important role in gene transcription and expression and can trigger production of inflammatory mediators [40], [41]. Baba-Moussa et al. suggested that calcium ion entry induced by PVL was the trigger of IL-8 production by PMNs [22]. In our study, however, LukG or LukH single components did not induce calcium ion entry, while triggering high levels of IL-8 production by PMNs. This suggests that LukG or LukH stimulated IL-8 production is independent of intracellular calcium ion concentration. LukG and LukH may bind cell surface proteins that lead to NF-κB activation, such as toll-like receptors (TLRs). There is some precedence for this, as LukS-PV triggers NF-κB activation through TLR2/CD14 [14] and HlgB induces NF-κB activation in a TLR-4 dependent pathway [13].

Genes for LukGH, γ-hemolysin and LukDE are present in the *S. aureus* genome, while genes for PVL are carried by phage and are found in 2–10% of circulating *S. aureus* strains [3]. Thus a *S. aureus* strain carrying genes for PVL (such as community-associated MRSA) can theoretically form 20 different toxin pairs (five S proteins × four F proteins). These multiple potential toxin pairings may broaden host specificity or provide additional pathogenic benefit. Other groups have characterized heterologous pairings of PVL, γ-hemolysin or LukDE components indicating the structure-function relationships amongst these BCLs [10], [19]. We sought to determine how LukGH contributes to this diversity of the staphylococcal BCL family. In all the assays we performed, including calcium flux and cytotoxicity in PMNs as well as hemolysis of RBCs, LukG did not form active pairings with heterologous S proteins nor did LukH form active pairings with heterologous F proteins (Table 2). This is in contrast to toxin pairs generated from PVL, γ-hemolysin and LukDE, all which display some activity on PMNs. The lack of cooperation by LukG or LukH with the other staphylococcal BCLs can be explained by significant sequence differences. Sequence homology of S components is ∼70% amongst LukS-PV, LukE, HlgA and HlgC, but only ∼30% with LukH. Similarly, the sequence homology of F components is ∼70% amongst LukF-PV, LukD and HlgB, but only about 30% with LukG. Ventura et al suggest the possibility of LukGH acting synergistically with PVL [11]. In a recent report looking at toxin-induced IL-1β release by macrophages, no synergistic activity was observed with PVL in combination with LukGH while combinations of PVL and other toxins including γ-hemolysin showed significant synergy in IL-1β release [9]. We did not directly examine cooperation or synergy of LukGH and PVL, however, our studies indicate that LukG and LukH do not have detectable activity when combined with any of the individual proteins of the other BCL families (Hlg, LukDE or PVL).

It is currently unclear what these observations mean in terms of active infection with *S. aureus*. The concentrations of proteins used in these assays is likely to be higher than that achieved in vivo. To our knowledge, bacterial strains that produce a single BCL component (e.g., only LukG or LukH) have not been identified. Thus, during infection with known *S. aureus* strains, these individual components would be expected to be present in similar amounts to their sister molecule and therefore lysis would be more likely than induction of inflammatory mediators. However, others have shown roles for single components LukS-PV or HlgB (termed LukF in the cited study) in stimulating host inflammatory responses at 280 nM and 300 nM, respectively [13], [14]. It is possible that there are strains that produce one defective protein unable to interact with the other component. Another possibility that might yield an imbalance or excess of a particular component would be more effective inactivation of a single component by host antibody responses. It is also possible that tissue diffusion of LukG differs from LukH and thus amounts of these proteins may be significantly different in certain sites. In these scenarios, induction of IL-8 would likely be preferred over cell lysis by LukGH. Further studies looking at activity of LukGH genetic variants and at *S. aureus* infection models would address some of these issues and may shed further light onto whether these observations have further implications in pathogenesis of staphylococcal infections.

In conclusion, these studies add to our understanding of this remarkable family of toxins produced by *S. aureus*. Our results indicate that LukGH is different from other staphylococcal BCLs in at least three ways. First, LukGH induced calcium ion entry into PMNs with slow and attenuated kinetics, which was qualitatively different from that induced by PVL, γ-hemolysin or LukDE. Second, LukG or LukH cannot make active heterologous pairs with PVL, γ-hemolysin or LukDE. Finally, and most uniquely, LukG and LukH single components are potent inducers of IL-8 production by PMNs. These findings suggest LukG and LukH are structurally and functionally different from other BCLs and that they may play important roles in inflammatory responses during *S. aureus* infection.
